# The Evolution of Stomach Acidity and Its Relevance to the Human Microbiome

**DOI:** 10.1371/journal.pone.0134116

**Published:** 2015-07-29

**Authors:** DeAnna E. Beasley, Amanda M. Koltz, Joanna E. Lambert, Noah Fierer, Rob R. Dunn

**Affiliations:** 1 Department of Biological Sciences, North Carolina State University, Raleigh, North Carolina, United States of America; 2 Department of Biology, Duke University, Durham, North Carolina, United States of America; 3 Department of Anthropology, University of Colorado, Boulder, Colorado, United States of America; 4 Department of Ecology and Evolutionary Biology, University of Colorado, Boulder, Colorado United States of America; 5 Cooperative Institute for Research in Environmental Sciences, University of Colorado, Boulder, Colorado, United States of America; Chengdu Institute of Biology, CHINA

## Abstract

Gastric acidity is likely a key factor shaping the diversity and composition of microbial communities found in the vertebrate gut. We conducted a systematic review to test the hypothesis that a key role of the vertebrate stomach is to maintain the gut microbial community by filtering out novel microbial taxa before they pass into the intestines. We propose that species feeding either on carrion or on organisms that are close phylogenetic relatives should require the most restrictive filter (measured as high stomach acidity) as protection from foreign microbes. Conversely, species feeding on a lower trophic level or on food that is distantly related to them (e.g. herbivores) should require the least restrictive filter, as the risk of pathogen exposure is lower. Comparisons of stomach acidity across trophic groups in mammal and bird taxa show that scavengers and carnivores have significantly higher stomach acidities compared to herbivores or carnivores feeding on phylogenetically distant prey such as insects or fish. In addition, we find when stomach acidity varies within species either naturally (with age) or in treatments such as bariatric surgery, the effects on gut bacterial pathogens and communities are in line with our hypothesis that the stomach acts as an ecological filter. Together these results highlight the importance of including measurements of gastric pH when investigating gut microbial dynamics within and across species.

## Introduction

Often, vertebrate stomach evolution is discussed in the context of the stomach’s role in chemically breaking down food and, specifically, denaturing proteins via pepsinogen and HCl [1]. The stomach clearly serves these purposes. However in light of our growing understanding of microbial symbionts’ role in human health, it is interesting to reassess the stomach’s additional role as an important barrier against pathogen entry into the gastrointestinal tract [2–3]. Here we consider the ecology of bird and mammal stomachs and, in the same light, medical interventions that alter human gastric pH and their implications for the human-microbe relationship.

Early studies of the human gut microbiome suggested that gut colonization was stochastic and transitory [4] and the microbiome at any particular moment was strongly influenced by recent colonists [5]. This pattern, if general, would have suggested a modest role for the human stomach in modulating the composition of the intestines. However, recent research in gastric health suggests that the pH environment of simple-stomached vertebrates serves a more prominent function as an ecological filter, capable, through its acidity, of killing microbial taxa that would otherwise colonize the intestines [2]. In this context, successful colonization would be infrequent. Recent studies show that in the absence of severe perturbation, the temporal variability in microbial composition of the human gut is less than the variability between individuals [6–8]. When major changes occur in healthy individuals, they often appear due to changes in the relative abundance of taxa rather than the arrival of new lineages [9]. More and more, data seem to suggest that species-specific communities in the human gut appear relatively resistant to perturbation [10–11], in large part because the acidic human stomach prevents frequent colonization of the gut by large numbers of food-borne microbes, regardless of whether they are beneficial or pathogenic.

While the literature on the human stomach, its acidity and the gut microbiome, seems to support the idea that stomach acidity evolved as a barrier to pathogen colonization, such an assertion makes full sense only in light of a broader comparative understanding of stomach acidity in birds and mammals [12–13]. Yet, while the idea that the stomach serves as a barrier to pathogens has often been discussed [14–16], no study appears to have formally compared the stomach pH in birds or mammals as a function of their biology in general or their likely exposure to foodborne pathogens in particular.

Because maintaining an acidic pH environment is costly, acidic stomachs should be present primarily in those cases where it is adaptive (or where it was adaptive in a recent ancestor). The cost of stomach acidity is twofold. The host must invest significant energy for both acid production and protecting the stomach from acid-related damage [17]. In addition, the acidity of the stomach may preclude, or at least make more difficult, chance acquisition of beneficial microbes. At the opposite extreme are those specialized herbivores in which stomach morphology is derived to include an alkaline chamber (forestomach or pre-saccus) that house microbes critical for fermenting a plant diet [18–22]. In these animals, an acidic stomach is not only of limited value (because the risk of foodborne pathogens in plant material is low), it may also remove those microbes that aid in the breakdown of plant material. Broadly then, we expect stomach acidity to mirror animal diets in ways that reflect pathogen risk. We expect that animals feeding on carrion will have the most restrictive filter, i.e. higher stomach acidity. Carrion has the potential to sustain high pathogen loads because the dead host’s body has stopped suppressing bacterial growth. Similarly, carnivores and omnivores would be expected to have higher stomach acidities than herbivores with specialized fermenting forestomachs because pathogens found in prey are more likely to be capable of infecting the predator than plant-associated microbes [23]. However, we would also expect the acidity of the carnivore and omnivore stomach to also depend on the phylogenetic distance between predator and prey. Pathogens are far more likely to be able to infect related hosts [23], such that a bird consuming an insect should face a lower risk of a foodborne infection than a bird consuming a bird. To test these hypotheses, we compare the stomach acidity of mammals and birds across a diversity of diet types.

In light of the results, we then revisit the ecology of the human stomach, its role as a filter and the likely consequences of this role within the context of modern human lifestyles and medical interventions. If stomach acidity acts as a strong filter, we expect that when acidity levels are reduced, the influence of diet-associated microbes on the intestinal microbiota will be greater. It is known that stomach acidity decreases with age and as a consequence of some medical treatments [24–26]. Thus, as acidity decreases and the filter’s effectiveness is reduced, we would expect to see increases in both the diversity of microbial lineages and pathogen loads in the gut. We also expect that animals, such as humans, with very acidic filters should be particularly predisposed to negative consequences of the loss of gut symbionts because the odds of chance re-colonization are low.

## Materials and Methods

### Vertebrate stomach

**Fig 1:**
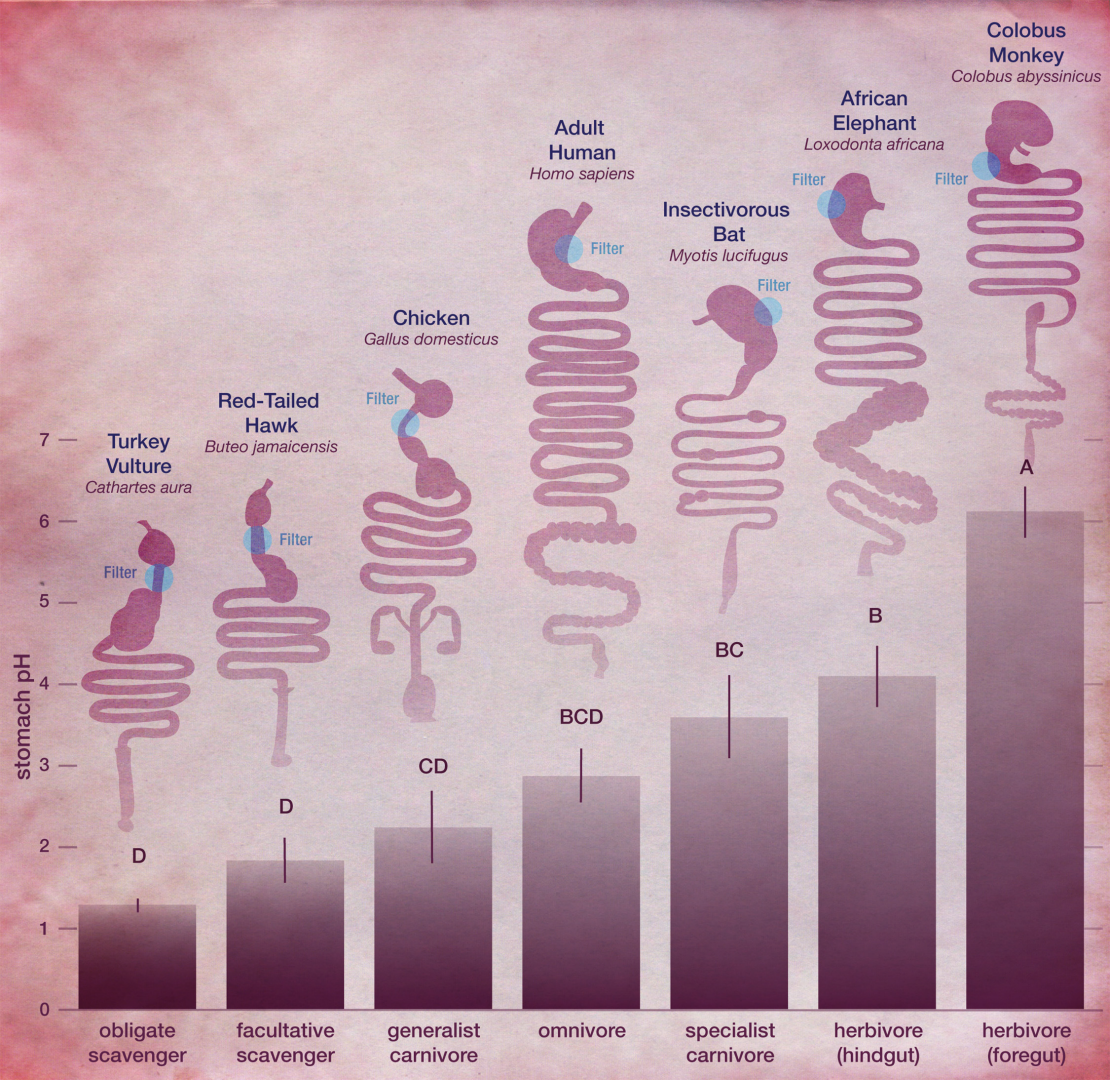
Comparison of stomach pH (mean ± S.E.) across trophic groups with gastrointestinal tracts of representative birds and mammals. Different letters above error bars represent statistically significant differences (P < 0.05) using ANOVA and Tukey-Kramer post-hoc test. Obligate scavengers (1.3 ± 0.08), facultative scavengers (1.8 ± 0.27), generalist carnivore (2.2 ± 0.44), omnivore (2.9 ± 0.33), specialist carnivore (3.6 ± 0.51), hindgut herbivore (4.1 ± 0.38) and foregut herbivore (6.1 ± 0.31).

Here we focus on two taxonomic groups, mammals and birds, in which the ecology of stomachs has been best studied. Within these taxa, we focus on the first chamber of the gastrointestinal tract, a chamber with different names depending on the organisms and context. In mammals, gastric acid production and temporary food storage both occur in the stomach. In birds, acid production occurs in the proventriculus and food storage occurs in the gizzard (Fig 1). We focus on the stomachs of mammals and, technically, the proventriculus of birds, but hereafter use the term “stomach” for simplicity. Stomachs vary greatly in their structural complexity and size among vertebrates [27], particularly mammals, yet in most of these cases, stomachs are the most acidic component of the digestive tract [28]. The exception to this pattern are forestomach-fermenting species in which microbial fermentation precedes digestion and absorption [22]. Mammalian herbivore clades can be characterized on the basis of where in the gastrointestinal tract most alloenzymatic (microbial) fermentation of dietary carbohydrate occurs. In foregut fermenters, microbes reside in one to several sections of a sacculated stomach. Among primates, only one lineage (subfamily Colobinae) has evolved this system, but analogous digestive strategies are found in several lineages of Artiodactyla as well as sloths, and kangaroos [18–22]. Among birds, only one species is known to rely on such a fermentation system (hoatzin, *Opisthocomus hoazin)* although microbes are housed in a specialized two-chambered crop, and not, technically, in the stomach [29]. Regardless of morphology, because communities of cellulolytic microorganisms and healthy fermentation occur most productively in an alkaline environment, the proximal portion of the foregut-fermenting stomach has a pH of approximately 5.5 to 7, while the distal portions have a pH of about 3. The need to maintain a particular pH in the forestomach no doubt influences feeding decisions: when the production of volatile fatty acids from fermentation exceeds absorption, the overabundance of acids can cause a drop in forestomach pH, resulting in a sometimes fatal affliction known as acidosis.

### Literature search

We searched gastrointestinal biology, animal physiology and avian physiology textbooks for measured stomach pHs. We also searched Web of Science, PubMed, Google Scholar and unpublished literature (i.e. dissertations, conference abstracts) for relevant data. Given its primary role in digestion, stomach pH has been measured in far fewer taxa than might be expected. For instance, to the best of our knowledge, no data on stomach pH exist for any hominoid other than humans, and surprisingly few data exist for primates more generally. Similarly, while it is wildly held that hyenas have “very acidic” stomachs, consultation of experts in hyenas and their diets were aware of no data that actually directly considered this assertion.

For those taxa for which data were available, we categorized animals by taxonomic group (bird or mammal), species and trophic group. For consistency, we assigned trophic group based on the animals’ natural feeding behavior (Table 1). A species was classified as an obligate scavenger if it fed primarily on carrion. Conversely, we defined facultative scavengers as species that are known to feed on carrion but not as a primary food source. We classified carnivores as a generalist if it fed indiscriminately on prey items or a specialist if the diet consisted primarily of a specific prey item (i.e. insects, fish). A species was categorized as an omnivore if it fed on both plants and animals. Within herbivores, we separated foregut fermenters from hindgut fermenters to account for the specialized fermentation strategy involving sacculated stomachs.

**Table 1:** Stomach pH data included in empirical analysis. Taxonomic class, order, species’ common and scientific names, trophic groups, stomach pH and reference(s). Fermentation strategy and specialized diets are indicated within trophic group category.

Class	Order	Common name	Scientific name	Trophic group	pH	Reference
Mammalia	Perissodactyla	shetland ponies	*Equus caballus var*	herbivore/hindgut	5.9	[48]
Mammalia	Monotremata	echidna	*Tachyglossus aculeatus*	specialist carnivore/Insect	6.8	[49]
Mammalia	Primates	colobus monkey	*Colobus polykomos*	herbivore/foregut	6.3	[50]
Mammalia	Artiodactyla	brocket deer	*Mazama sp*.	herbivore/foregut	5.5	[51]
Mammalia	Primates	cynomolgus monkey	*Macaca fascicularis*	omnivore	2.1	[52]
Mammalia	Perissodactyla	rhino	*Diceros bicornis*	herbivore/hindgut	3.3	[53]
Mammalia	Proboscidea	elephant	*Loxodonta africana*	herbivore/hindgut	3.3	[53]
Mammalia	Artiodactyla	hippo	*Hippopotamus amphibius*	herbivore/hindgut	4.4	[53–54]
Mammalia	Edentata	sloth	*Choloepus sp*.	herbivore/foregut	7.4	[54]
Mammalia	Artiodactyla	collared peccary	*Tayassu pecari*	herbivore/foregut	5.8	[54]
Mammalia	Primates	skyes monkey	*Cercopithecus mitis*	omnivore	3.4	[55]
Mammalia	Primates	baboon	*Papio cynocephalus*	omnivore	3.7	[55]
Mammalia	Primates	langur monkey	*Presbytis cristatus*	herbivore/foregut	5.9	[56]
Mammalia	Marsupialia	macropodid	*Macropodidade*	herbivore/foregut	6.9	[57]
Aves	Galliformes	chicken	*Gallus gallus domesticus*	specialist carnivore/Insect	3.7	[58–59]
Mammalia	Primates	humans	*Homo sapiens*	omnivore	1.5	[60–61]
Aves	Falconiformes	grey falcon	*Falco rusticolus*	facultative scavenger	1.8	[62]
Aves	Falconiformes	peregrine falcon	*Falco peregrinus*	facultative scavenger	1.8	[62]
Aves	Falconiformes	red tailed hawk	*Buteo jamaicensis*	facultative scavenger	1.8	[62]
Aves	Falconiformes	swainson's hawk	*Buteo swainsoni*	facultative scavenger	1.6	[62]
Aves	Strigiformes	snowy owl	*Nyctea scandiaca*	generalist carnivore	2.5	[62]
Aves	Falconiformes	bald eagle	*Haliaetus leucocephalus*	facultative scavenger	1.3	[62]
Aves	Strigiformes	great horned owl	*Bubo virginianus*	generalist carnivore	3.1	[62]
Mammalia	Carnivora	ferret	*Mustela putorius furo*	generalist carnivore	1.5	[63]
Aves	Procellariiformes	wandering albatross	*Diomedea exulans*	obligate scavenger	1.5	[64]
Aves	Sphenisciformes	gentoo penguin	*Pygoscelis papua*	specialist carnivore/Fish	2.5	[64]
Aves	Sphenisciformes	magellanic penguin	*Spheniscus magellanicus*	specialist carnivore/Fish	2.3	[64]
Aves	Accipitriformes	white backed vulture	*Gyps africanus*	obligate scavenger	1.2	[65]
Mammalia	Marsupialia	southern hairy nosed wombat	*Lasiorhinus latifrons*	herbivore/hindgut	3.3	[66]
Mammalia	Marsupialia	woylie brush tailed bettong	*Bettongia penicillata*	herbivore/hindgut	2.8	[67]
Mammalia	Rodentia	beaver	*Castor canadensis*	herbivore/hindgut	1.7	[68]
Mammalia	Primates	howler monkey	*Alouatta palliata*	herbivore/hindgut	4.5	[69]
Mammalia	Cetacea	bottlenose dolphins	*Tursiops truncatus*	specialist carnivore/Fish	2.3	[70]
Mammalia	Marsupialia	quokka	*Setonix brachyurus (Quoy & Gaimard)*	herbivore/hindgut	7.4	[71]
Mammalia	Cetacea	minke whale	*Balaenoptera acutorostrata*	specialist carnivore/Fish	5.3	[72]
Mammalia	Primates	silver leafed monkey	*Tracypithecus cistatus*	herbivore/foregut	5.9	[73]
Aves	Pelecaniformes	american bittern	*Botaurus lentiginosus*	facultative scavenger	1.7	[74]
Mammalia	Marsupialia	possum	*Trichosurus vulpecula*	facultative scavenger	1.5	[75]
Mammalia	Artiodactyla	ox	*Bos sp*.	herbivore/foregut	4.2	[76]
Mammalia	Artiodactyla	sheep	*Ovis aries*	herbivore/foregut	4.7	[76]
Mammalia	Perissodactyla	horse	*Equus ferus caballus*	herbivore/hindgut	4.4	[76]
Mammalia	Rodentia	gerbil	*Gerbillinae sp*.	herbivore/hindgut	4.7	[76]
Mammalia	Rodentia	guinea pig	*Cavia porcellus*	herbivore/hindgut	4.3	[76]
Mammalia	Rodentia	hamster	*Cricetinae sp*.	herbivore/hindgut	4.9	[76]
Mammalia	Lagomorpha	rabbit	*Oryctolagus cuniculus*	herbivore/hindgut	1.9	[76]
Mammalia	Primates	crab-eating macaque	*Macaca irus*	omnivore	3.6	[76]
Mammalia	Rodentia	mouse	*Mus musculus*	omnivore	3.8	[76]
Mammalia	Rodentia	rat	*Rattus norvegicus*	omnivore	4.4	[76]
Mammalia	Artiodactyla	domesticated pig	*Sus scrofa domesticus*	omnivore	2.6	[76–77]
Mammalia	Carnivora	dog	*Canis lupus familiaris (beagle)*	facultative scavenger	4.5	[78–81]
Mammalia	Carnivora	cat	*Felis catus*	generalist carnivore	3.6	[78; 82]
Mammalia	Chiroptera	common pipistrelle bat	*Pipistrellus pipistrellus*	specialist carnivore/Insect	5.1	[83–84]
Aves	Sphenisciformes	king penguins	*Aptenodytes patagonicus*	specialist carnivore/Fish	2.9	[85]
Mammalia	Artiodactyla	guanaco	*Lama guanicoe*	herbivore/foregut	7.3	[86]
Mammalia	Artiodactyla	llama	*Lama glama*	herbivore/foregut	7	[86]
Mammalia	Rodentia	porcupine	*Erethizon dorsatum*	herbivore/hindgut	4.5	[87]
Mammalia	Artiodactyla	camel	*Camelus sp*.	herbivore/foregut	6.4	[88]
Aves	Strigiformes	barn owl	*Tyto alba*	facultative scavenger	1.3	[89–90]
Aves	Strigiformes	little owl	*Athene noctua*	facultative scavenger	1.3	[90]
Aves	Charadriformes	black-headed gull	*Larus ridibundus*	facultative scavenger	1.5	[90]
Aves	Falconiformes	common kestrel	*Falco tinnunculus*	generalist carnivore	1.5	[90]
Aves	Charadriformes	common pied oystercatcher	*Haematopus ostralegus*	generalist carnivore	1.2	[90]
Aves	Accipitriformes	common buzzard	*Buteo buteo*	obligate scavenger	1.1	[90]
Aves	Passeriformes	carrion crow	*Corvus corone*	obligate scavenger	1.3	[90]
Aves	Gruiformes	common moorhen	*Gallinula chloropus*	omnivore	1.4	[90]
Aves	Passeriformes	common starling	*Sturnus vulgaris*	specialist carnivore/Insect	2	[90]
Aves	Anseriformes	mallard duck	*Anas platyrhynchos*	omnivore	2.2	[58; 62; 90]
Aves	Suliformes	great cormorant	*Phalacrocorax carbo carbo*	specialist carnivore/Fish	3	[90–91]

We calculated a mean pH for the entire stomach if values were presented for multiple locations such as the fundus, body and pyloric regions. If studies provided both baseline and post-feeding values, we used the baseline pH. When not fasting, pH may vary depending on factors including diet and time since feeding.

We used a general linear model approach followed by a Tukey-Kramer post-hoc test to assess differences in stomach pH as a function of trophic category using PROC GLM in SAS 9.3 (SAS Institute, Cary, NC, USA).

## Results

In total, our literature search yielded data on 68 species (25 birds and 43 mammals) from seven trophic groups (Table 1). A general linear model based on diet explained much of the variation in the stomach pH (R^2^ = 0.63, F_1,6_ = 17.63, p < 0.01). The trophic groups that were most variable in terms of their stomach pH were omnivores and carnivores that specialize in eating insects or fish.

Our hypothesis was that foregut-fermenting herbivores and animals that feed on prey more phylogenetically–distant from them would have the least acidic stomachs. Tukey-Kramer comparisons indicated that scavengers (both obligate and facultative) had significantly higher stomach acidities compared to herbivores (both foregut and hindgut) and specialist carnivores feeding on phylogenetically distant prey. Specifically, foregut-fermenting herbivores had the least acidic stomachs of all trophic groups while omnivores and generalist carnivores, with more intermediate pH levels, were not distinguishable from any other group (Fig 1).

## Discussion

Based on the available data, our analysis illustrates a general pattern in which species feeding on carrion and animals have significantly higher stomach acidities compared to species feeding on insects, leaves, or fruit. On their own, the patterns are in line with the hypothesis that one role of the stomach is to inhibit microbial entry into the gut, though these patterns might also be explained by other phenomena. Carnivores need more acidic stomachs in order to lyse the protein in their meat-based diets. For example, secretion of pepsinogen and its activation to pepsis in the stomach is modulated by an acid pH (2–4) [30]. Also, activity of proteases in a simple acid stomach depends on an acidic environment (pH 2–4) [31]. However, while this might explain differences between predators and herbivores, it does not account for the very high acidity in the stomachs of scavengers, especially considering that the meat consumed by scavengers is not likely to be much harder to digest than that of predators. We suggest that these scavengers rely on the high acidity of their stomach to prevent colonization of their guts by foodborne pathogens [32]. Omnivores and piscivores were most variable in stomach acidities, which is to be expected as both diets differ greatly from species to species. Insectivores may use diverse means to digest insect chitin, with acidity playing a role in some but not other cases.

### The special case of herbivory

Carrion feeding imposes one sort of constrain on the ecology of the gut, an increase in the potential for pathogens. Herbivory imposes another, the need to digest plant material refractory to enzymatic digestion (cellulose and lignin). In order to digest these compounds, herbivores rely disproportionately on microbial processes [33]. Different regions of the gastrointestinal tract (either rumen, caecum or in the case of the hoatzin a folded crop) function primarily as fermentation chambers. Thus, a challenge with fermentative guts is favoring those microbes that are useful for digestion while reducing the risk of pathogen entry into the gut. We suggest that because the threat of microbial pathogens is relatively low on live leaves (although see [34]), herbivores can afford to maintain a chamber that is modestly acidic and therefore less restrictive to microbial entry. However, we find several interesting exceptions to this generality. Beavers, which are known to store food caches underwater where there is a high risk of exposure to a protozoan parasite *Giardia lamblia*, have very acidic stomachs. The high stomach acidity may have evolved to manage this prevalent environmental pathogen [35]. The other herbivore in our dataset with a very acidic stomach is the rabbit, which provides an interesting example of a behavioral modification of the stomach environment. Rabbits are known to engage in frequent coprophagy which allows them re-inoculate themselves with microbes [36]. The specialized soft pellets that house microbes also reduce the stomach acidity creating an environment suitable for fermentation [37].

### Human evolution and stomach pH

It is interesting to note that humans, uniquely among the primates so far considered, appear to have stomach pH values more akin to those of carrion feeders than to those of most carnivores and omnivores. In the absence of good data on the pH of other hominoids, it is difficult to predict when such an acidic environment evolved. Baboons (*Papio* spp) have been argued to exhibit the most human–like of feeding and foraging strategies in terms of eclectic omnivory, but their stomachs–while considered generally acidic (pH = 3.7)–do not exhibit the extremely low pH seen in modern humans (pH = 1.5) [38]. One explanation for such acidity may be that carrion feeding was more important in humans (and more generally hominin) evolution than currently considered to be the case (although see [39]). Alternatively, in light of the number of fecal-oral pathogens that infect and kill humans, selection may have favored high stomach acidity, independent of diet, because of its role in pathogen prevention.

### The special risk to juvenile and elderly humans

If, in carnivores and carrion-feeders, the stomach’s role is to act as an ecological filter then we would also expect to see higher microbial diversity and pathogen loads in cases where stomach pH is higher. We see evidence of this in age-related changes in the stomach. Baseline stomach lumen pH in humans is approximately 1.5 (Table 1). However, premature infants have less acidic stomachs (pH > 4) and are susceptibility to enteric infections [40]. Similarly, the elderly show relatively low stomach acidity ([41], pH 6.6 in 80% of study participants) and are prone to bacterial infections in the stomach and gut [42]. It is important to note that these differences may be related to differences in the strength of the immune system however we argue here that the stomach needs more consideration when studying these patterns.

### Consequences for medical interventions that influence stomach pH

In addition to natural variation, stomach pH is also affected by some medical interventions, several of which are increasingly common. In gastric bypass weight loss surgery, roughly 60 percent of the stomach is removed. A consequence of this procedure is an increase in gastric pH levels that range from 5.7 to 6.8. We would predict that the intestines of those individuals who have had gastric bypass surgery should be more likely to experience microbial overgrowth, a pattern that is supported by recent work [25]. We see similar patterns in other clinical cases such as oesophagitis in which treatment involves the use of proton-pump inhibitors and celiac disease where delayed gastric emptying is associated with reduced acidity [43–45]. More generally, we predict that individuals undergoing interventions that reduce the acidity of their stomachs will be at long term increased risk of gastrointestinal pathogens. However, this risk may be reduced if such individuals tend to avoid foods in which pathogen risk is elevated, which include (as for birds and mammals more generally) foods that resemble carrion (raw fish, raw mammal meat, etc…), and perhaps even meat in general. Thus, one might expect the optimum pH for humans to change depending on changes in eating habits.

### The human stomach and the loss of mutualistic microbes

In general, stomach acidity will tend to filter microbes without adaptations to an acidic environment. Such adaptations include resistant cell walls, spore-forming capabilities or other traits that confer tolerance to high acidities and rapid changes in pH conditions. We’ve considered the role of the stomach as a pathogen barrier within the context of human evolution. Another potential consequence of high stomach acidity, when considered in light of other primates and mammals, is the difficulty of recolonization by beneficial microbes. A large body of literature now suggests that a variety of human medical problems relate to the loss of mutualistic gut microbes, whether because those mutualists failed to colonize during hyper-clean C-section births [46] or were lost through use of antibiotics [47], or other circumstances. The pH of the human stomach may make humans uniquely prone to such problems. In turn, we might expect that, among domesticated animals, that similar problems should be most common in those animals that, like us, have very acidic stomachs.

## Conclusion

We demonstrate that stomach acidity increases with the risk of food-borne pathogen exposure and propose that the stomach plays a significant role as an ecological filter and thus a strong selection factor in gut microbial community structure and primate evolution in particular. In light of modern lifestyle changes in diet, hygiene and medical interventions that alter stomach pH, we suggest that stomach acidity in humans is a double-edged sword. On one hand, the high acidity of the human stomach prevents pathogen exposure but it also decreases the likelihood of recolonization by beneficial microbes if and when they go missing. However, in those cases where acidity is reduced, the gut is more likely to be colonized by pathogens. Though it is widely discussed in both the medical and ecological literature, data on pH are actually very scarce. Thus, to fully understand the patterns highlighted here more detailed studies on the gut microbiota across stomach acidities and diet are required.
